# 3D culture of Her2+ breast cancer cells promotes AKT to MAPK switching and a loss of therapeutic response

**DOI:** 10.1186/s12885-016-2377-z

**Published:** 2016-06-01

**Authors:** Sharath Gangadhara, Chris Smith, Peter Barrett-Lee, Stephen Hiscox

**Affiliations:** School of Pharmacy and Pharmaceutical Sciences, Cardiff University, Redwood Building, CF10 3NB Cardiff, UK; Velindre Cancer Centre, Whitchurch Road, Cardiff, UK

**Keywords:** 3D culture, Her2+ breast cancer, MAPK, AKT, Therapeutic response

## Abstract

**Background:**

The Her2 receptor is overexpressed in up to 25 % of breast cancers and is associated with a poor prognosis. Around half of Her2+ breast cancers also express the estrogen receptor and treatment for such tumours can involve both endocrine and Her2-targeted therapies. However, despite preclinical data supporting the effectiveness of these agents, responses can vary widely in the clinical setting. In light of the increasing evidence pointing to the interplay between the tumour and its extracellular microenvironment as a significant determinant of therapeutic sensitivity and response here we investigated the impact of 3D matrix culture of breast cancer cells on their therapeutic sensitivity.

**Methods:**

A 3D Matrigel-based culture system was established and optimized for the growth of ER+/Her2+ breast cancer cell models. Growth of cells in response to trastuzumab and endocrine agents in 3D culture versus routine monolayer culture were assessed using cell counting and Ki67 staining. Endogenous and trastuzumab-modulated signalling pathway activity in 2D and 3D cultures were assessed using Western blotting.

**Results:**

Breast cancer cells in 3D culture displayed an attenuated response to both endocrine agents and trastuzumab compared with cells cultured in traditional 2D monolayers. Underlying this phenomenon was an apparent matrix-induced shift from AKT to MAPK signalling; consequently, suppression of MAPK in 3D cultures restores therapeutic response.

**Conclusion:**

These data suggest that breast cancer cells in 3D culture display a reduced sensitivity to therapeutic agents which may be mediated by internal MAPK-mediated signalling. Targeting of adaptive pathways that maintain growth in 3D culture may represent an effective strategy to improve therapeutic response clinically.

**Electronic supplementary material:**

The online version of this article (doi:10.1186/s12885-016-2377-z) contains supplementary material, which is available to authorized users.

## Background

Breast cancer is the most frequently diagnosed female cancer globally and is the leading cause of cancer death in women [1]. In the UK, the current lifetime risk of developing the disease for women is currently 1 in 8 [2, 3]. Overexpression or amplification of the Her2 gene product occurs in around 20 % of all breast cancers and around half of Her2+ tumours will also co-express the estrogen receptor (ER) [4]. Despite the effectiveness of endocrine and Her2-targeted therapies for such tumours in pre-clinical, two-dimensional models, the clinical response to these treatments can vary greatly with therapeutic resistance a limiting factor; resistant tumours frequently present as metastases with associated poor prognosis highlighting the need for more effective treatments in the early phases of the disease.

Increasing evidence now points to the interplay between the tumour and its surrounding microenvironment as a significant determinant of therapeutic sensitivity and response [5, 6] with tumour-stroma interactions demonstrated to influence tissue response to ionizing radiation [7], chemotherapeutics and more recently targeted agents [8, 9]. The influence of stroma on the therapeutic response to cytotoxic drugs has been investigated through studies using matrix-rich 3D culture environments where tumour cells grown in such a manner exhibit resistance to doxorubicin compared to responses in traditional 2D culture [10]. Furthermore, the migration of fibrosarcoma cells in 2D culture is decreased by doxorubicin chemotherapy whereas this effect is completely abolished when grown in the context of a 3D collagen-rich matrix [11].

Tumour cell-extracellular matrix interactions may attenuate drug response through alterations in internal signalling pathways, possibly as a result of integrin activation. For example, matrix-induced β-1 integrin activation results in suppression of chemotherapy-induced apoptosis and enhanced tumourigenecity [12] and promotes resistance to cisplatin [13]. The interaction of cells with laminin, mediated through a range of alpha and beta integrins, is also able to enhance tumourigenecity and decrease sensitivity to cytotoxic agents [14]. Importantly, clinical studies have shown that ECM composition of tumour correlates with lack of clinical response to chemotherapy and reduced overall survival [15, 16]. Thus a better understanding of how tumours interact with their surrounding microenvironment is crucial for the development of more effective clinical treatment strategies. Here we have investigated the impact of the extracellular matrix on the therapeutic response and signaling pathway activity of ER+/Her2+ breast cancer cells with a view to identifying potential targets to improve therapeutic response.

## Methods

### Antibodies/Reagents

Routine cell culture reagents (RPMI 1640 media, Foetal Calf Serum (FCS), 3-(4,5-dimethylthiazol-2-yl)-2,5-diphenyltetrazolium bromide (MTT), Trypsin/EDTA, Amphotericin B (Fungizone), penicillin/streptomycin) were purchased from Invitrogen (Paisley, UK). Basement membrane matrix (Matrigel) and BD Cell Recovery Solution (Matrisperse) were obtained from BD Biosciences (supplied by VWR International Ltd, UK). The MEK inhibitor, U0126, and AKT inhibitor, MK-2206 2HCL, were from Promega Uk and Stratech Scientific Ltd, UK respectively. Enhanced chemiluminescence Supersignal® Western blotting detection reagents were purchased from Pierce and Warriner Ltd (Cheshire, UK). Antibodies recognizing total and phospho forms of Akt, MAPK, Erk1/2 and erbB2 were from Cell Signaling Technology (MA, USA); anti-GAPDH, anti-β-actin and secondary HRP-conjugated antibodies were from Sigma-Aldrich (Poole, Dorset, UK). The total-ER (clone 6 F11) mouse anti-human primary antibody was from NovoCastra.

### Cell lines and reagents

Two ER+/Her2+ cell models, BT474 and MDAMB361, were obtained from ATCC (American Type Culture Collection) and routinely maintained in RPMI supplemented with 10 % FCS, penicillin (100units/ml), streptomycin (100ug/ml) and amphotericin B (2.5ug/ml). Experiments utilising endocrine agents were performed in steroid-depleted culture conditions (phenol red-free RPMI containing 5 % charcoal-stripped FCS and antibiotics as above).

### Measurement of cell growth in 2D cell culture

Cells were harvested using trypsin/EDTA and reseeded into 48-well plates at a density of 20,000 cells/well in fresh media containing treatments as indicated. Cells were allowed to grow for 10 days with medium changes every 3 days. At the end of the experiment, the medium was removed and 0.5 ml of trypsin/EDTA was added to each well. Once the cells were in suspension, cells were drawn into a 5 ml syringe through a 25G needle three times to obtain a single-cell suspension. The wells were then washed with 0.5 ml of fresh Isoton II solution and this was then drawn up into the syringe. This final wash was repeated twice to give a total volume of 2 mls in the syringe. The solution was then added to 8 mls of Isoton II solution in a counting vial to make up a volume of 10mls. Cells were then counted (three counts per well) using a Coulter™ Multisizer II.

### Three-dimensional (3D) cell culture

For analysis of cellular growth in 3D culture, we used a modified version of the ‘3D on-top’ assay reported by Lee et al. [17] optimised for the cell lines under test here (Additional file 1: Figure S1A). Using this method allowed for the imaging of cell colonies in a single plane and cellular retrieval for counting assays. The 3D culture protocol was performed as follows: The wells of a 48-well plate were pre-coated in phenol red-free Matrigel (80ul/well) and incubated at 37 °C for 30 min to allow gel formation. Cells were harvested using trypsinisation and pelleted by centrifugation at 115 g before resuspension in fresh media and seeding into Matrigel-coated at a final concentration of 0.20 × 10^5^ cells/cm^2^. The cells were allowed to settle and attach to the Matrigel for 30 min at 37 °C following which they were overlaid with 1.2 mls of fresh media containing 10 % (v/v) Matrigel.. Cell cultures were maintained for up to 10 days, replacing the Matrigel-medium mixture every 3 days.

### Recovery of cells from 3D culture for experimental analysis

Cells were recovered from the 3D cultures for counting, immunocytochemical analysis or Western blotting using a modification of a previously reported method [18] and Additional file 2: S1B. Briefly, the medium was removed and each well was washed 3x with ice cold PBS. 10ul matrisperse (BD Biosciences) was added to each well and well contents gently recovered by scraping with a 1 ml syringe plunger. Recovered cells, along with two further well washings with matrisperse, were collected in universal tubes and left on ice for 60 min. At this stage, the cells extracted from 3D matrix are still in colonies and could be processed for cellblocks and immunocytochemistry. For cell counting and immunoblotting the following steps were performed. Universal tubes were spun at 5000 rpm for 5 min and the supernatant discarded. The cell pellets were re-suspended in 0.5 ml of trypsin/EDTA and incubated at 37 °C for 5 min prior to the addition of 1.5 ml of fresh Isoton II solution. This solution was drawn into a 5 ml syringe through a 25G needle three times to obtain a single-cell suspension. The solution was then added to 8 mls of Isoton II solution in a counting vial to make up a volume of 10mls. Cells were then counted using a Coulter™ Multisizer II. At least three counts were taken from each well.

### Western blotting

Cells were grown as monolayers or as 3D cultures in 35 mm dishes ± treatments then washed twice with ice-cold PBS and lysed with Triton-X100 lysis buffer (sodium orthovandate 2 mM, phenylmethylsulfonyl fluoride 1 mM, sodium fluoride 25 mM, sodium molybdate 10 mM, phenylarsine 20uM, and leupeptin 10ug/ml and aprotinin 8ug/ml in 50mMTris-HCL, pH8.0 containing 0.1 % TX-100). Cells were then collected using a cell scraper and transferred to a 1.5 ml micro-centrifuge tube, incubated on ice for 15 min before centrifugation at 13000 rpm, 15 min, 4 °C. For cells growing in 3D culture. the matrigel was first depolymerized using matrisperse prior to addition of lysis buffer. The supernatants were removed and stored at −20 °C until required. After protein determination (BioRad DC protein assay kit), equal amounts of protein were incubated with 2x sample loading buffer (4 % SDS, 10 % 2-mercaptoethanol, 20 % glycerol, 0.004 % bromophenol blue in 0.125 M Tris–HCl pH 6.8) and heated to 100C for 5 min. Cell lysates, together with molecular weight standards, were then separated on 8 % gels using SDS-PAGE. After transfer onto nitrocellulose membranes and blocking with 5 % milk (in TBS), membranes were probed for specific target proteins using appropriate antibodies.

### Preparation of cell pellet blocks for immunostaining

2D and 3D cultures of BT474 and MDAMB361 cultures were prepared and grown in 35 mm dishes ± treatments as indicated for 7 days. For standard (2D) cultures the media was aspirated and the cells gently removed using a cell scraper and re-suspended in phenol red RPMI. For 3D cultures, cell colonies were dissociated from the Matrigel™ Matrix using BD cell recovery solution in order to detach colonies whilst retaining their three-dimensional integrity.

The cells from 2D and colonies from 3D were then centrifuged at 1000 rpm for 5 min and fixed immediately in 4 % formaldehyde/PBS (1 hr room temp). Cells were then transferred to a Eppendorfs where they were allowed to settle under gravity for a further 50 min (in 4 % formaldehyde/PBS). For each sample, the supernatant was removed and a 1:1 ratio of molten 12 % Noble agar was added to the cell suspension. This was quickly transferred by Pasteur pipette into an inverted 2 ml syringe with its tip cut off, and clamped in a retort stand which functioned as a mould. The agar was left overnight to set. A cell plug was extruded from the syringe and cut into 5 mm sections placed into a standard Histo-TEK® cassette. These were fixed for a further 2 h in a 4 % formaldehyde/PBS solution.

The pellets were then dehydrated in a series of graded ethanol solutions (10 to 100 % v/v) for 45 min each (overnight for 70 %), cleared in Xylene for 2 h, left in a molten paraffin wax bath for 2 h (under vacuum for 30 min) before being embedded in paraplast medium to create a block. At least three pellets were assembled into each block which was subsequently sectioned at 5 μm to check integrity and density of cells in the pellets using H&E staining.

### Immunocytochemical staining

A 3 % aqueous solution of hydrogen peroxide was added to the sections for 5 min to block endogenous peroxidases following which sections were washed in PBS for a further 5 min. Antigen retrieval was performed by pressure-cooking sections in 2 l, 0.01 M Sodium Citrate Buffer (pH6) for 2 min. Slides were cooled under running tap water for 10 min then washed in PBS for 5 min.

For measurement of the total-ER, erbB2 and Ki67, sections were blocked with 20 % Normal Human Serum (ER) or 1 % BSA (erbB2) in PBS for 10 min prior to incubation with primary antibodies (dilutions in PBS were ER:1/80 and erbB2:1/350) for 1 h. After washing in PBS, a peroxidase labelled secondary antibody (Dako Mouse EnVision) was added and sections incubated for 2 h at room temperature. Slides were washed in PBS before addition DAB (Dako) for 10 min and rinsing in distilled water. Sections were counterstained with 0.5 % methyl green, allowed to air dry and then mounted.

For the Ki67 assay, a modified protocol was performed. Antigen retrieval was performed by microwaving sections in 1 l Citric Acid Buffer (2.1 g/l, pH 6) for 30 min at 560 W and cooling slides under running tap water for 10 min. The primary antibody was applied (Dako MIB (M7240) at 1/50 dilution in 0.1 % BSA/PBS) for 2 h, room temperature. Washing and secondary antibody incubation was performed as for the ER assay above. Ki67 stained sections were counterstained using 4 % haematoxylin.

### Statistical analysis

Comparisons of treatment effects on cell growth were performed using a paired *t*-test. Data from the cell proliferation assays were analyzed using GraphPad Prism (GraphPad Software Inc., San Diego, CA) to calculate the concentration of drug required for 50 % inhibition of cell growth (GI50) by nonlinear regression curve fitting with sigmoidal dose response (variable-slope) parameter.

## Results

### Breast cancer cells growing in 3D culture retain characteristics of 2D cultures

**Fig. 1:**
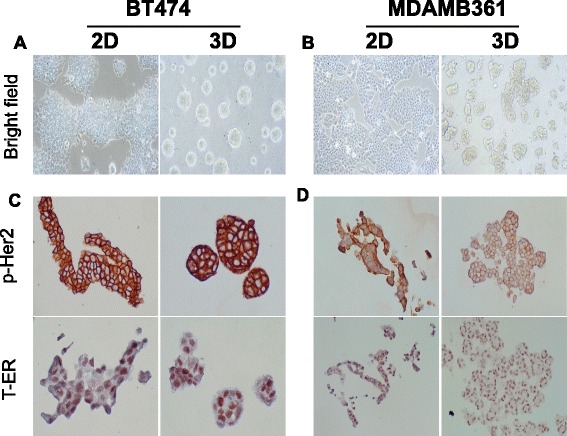
Breast cancer cells growing in 3D culture retain characteristics of 2D cultures. Bright field images of BT474 (**a**) and MDAMB361 (**b**) cells in 2D and 3D culture revealed that cells growing in 3D culture formed tightly packed spherical aggregates with a rounded (BT474) or grape-like (MDAMB361) appearance compared to 2D monolayers growth. Immunocytochemical staining of cellular receptors namely the Her2 and ER did not reveal any loss or change in cellular localization when cells were cultured in 3D compared with 2D (**c**, **d**)

In this study we set out to investigate the effects of 3D culture in a matrix-enriched environment on the therapeutic sensitivity of two ER+/Her2+ breast cancer cell models. After establishing an appropriate in vitro 3D culture system, we first explored whether 3D culture had any effect on the morphological appearance of the cell lines. Bright field images of BT474 (Fig. 1a) and MDAMB361 (Fig. 1b) cells in 2D and 3D culture revealed that cells growing in 3D culture formed tightly packed spherical aggregates with a rounded (BT474) or grape-like (MDAMB361) appearance compared to 2D monolayers growth.

We subsequently wished to explore whether 3D culture affected expression of cellular receptors that are the key therapeutic targets in these breast cancer models namely the Her2 and ER. Immunocytochemical staining of these markers did not reveal any loss or change in cellular localization when cells were cultured in 3D compared with 2D (Fig. 1c, d).

**Fig. 2:**
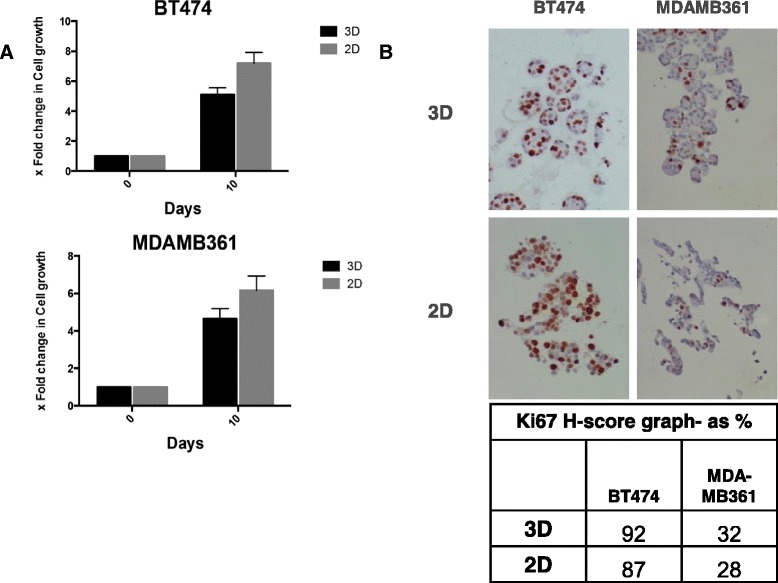
Basal growth rate of BT474 and MDAMB361 cells in 3D and 2D cultures. The basal growth of both cell lines (BT474 and MDAMB361) in control medium over a 7-day period in the 3D and 2D contexts were measured using coulter counting (**a**). There was a modest reduction in growth rate in 3D culture which was not statistically significant. Growth data by coulter counting was further validated by assessing expression of Ki67 (**b**). Again, no significant differences were observed between cells growing in 2D versus 3D culture

We then measured the basal growth of both cell lines in control medium over a 7-day period in the 3D and 2D contexts using coulter counting (Fig. 2a). Growth rates of cell lines cultures in 2D and 3D appeared very similar and, whilst there was a modest reduction in growth rate in 3D culture, this was not statistically significant. Growth data by coulter counting was further validated by assessing expression of Ki67 (Fig. 2b). Again, no significant differences were observed between cells growing in 2D versus 3D culture.

### Growth of ER+/Her2+ breast cancer cells in 3D culture attenuates their response to endocrine agents and trastuzumab

**Fig. 3:**
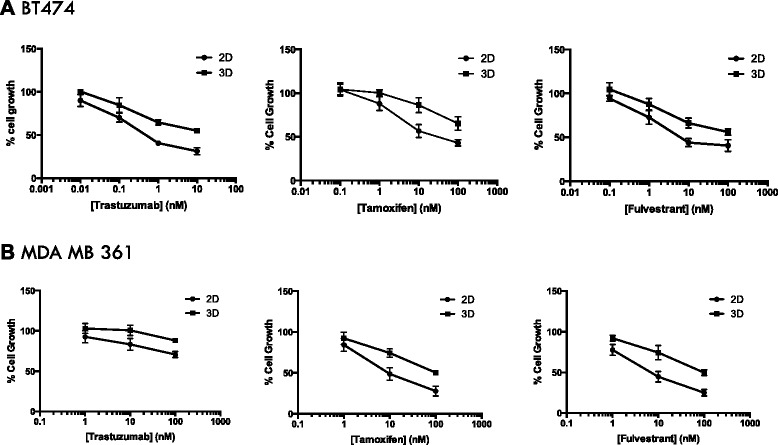
3D culture attenuates response to tamoxifen, fulvestrant and trastuzumab. BT474 (**a**) and MDAMB361 (**b**) cells were grown in 2D (SQUARE) or 3D (CIRCLE) cultures in the presence or absence of tamoxifen, fulvestrant or trastuzumab for 7 days after which cell number was assessed by Coulter counting. For both cell lines, culture in 3D conditions attenuated their response to endocrine agent or trastuzumab. Plots are mean cell growth ± SD

Studies have suggested that the 3D microenvironment may contribute to loss of chemosensitivity. To begin to explore this in the context of ER+/Her2+ breast cancer, BT474 and MDAMB361 cells were grown in 2D monolayers or 3D cultures and exposed to a range of concentrations of tamoxifen, fulvestrant or trastuzumab for 7 days after which cell numbers were counted. Whilst both cell lines exhibited a dose-dependent inhibition of cell growth in response to these agents in 2D culture conditions, the effects of endocrine agents and trastuzumab on cell growth was significantly reduced in 3D culture (Fig. 3a, b). IC50 values for each agent in 2D and 3D culture were calculated (Table 1) which again demonstrated that sensitivity was lost in 3D culture conditions.

**Table 1:** IC50 data for tamoxifen, fulvestrant and trastuzumab in 2D versus 3D culture conditions

Mean GI 50 (nM)						
Cell line	Trastuzumab		Tamoxifen		Fulvestrant	
	3D	2D	3D	2D	3D	2D
BT474	13	1.7	>100	14.5	100	9
MDAMB361	>100	>100	88	9	76	8.3

Cell growth data from Fig. 3 were analysed using GraphPad to obtain IC50 values. These were higher for both cell lines for each agent when cultured in 3D

### Combination treatment of ER+/Her2+ cell lines using endocrine agent and trastuzumab is attenuated in 3D culture

**Fig. 4:**
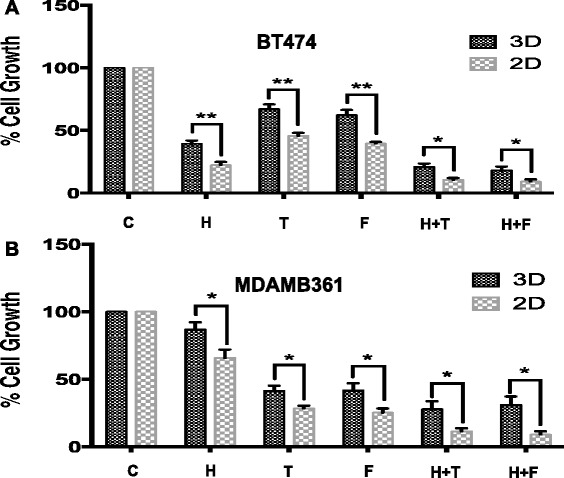
3D culture attenuates the therapeutic response of ER + Her2+ cells. BT474 (**a**) and MDAMB361 (**b**) cells were grown for 10 days as 2D monolayers or as 3D cultures in Matrigel and their proliferative response to trastuzumab and endocrine monotherapy or their combination assessed using coulter counting. The growth inhibitory effects of trastuzumab and endocrine therapy on day 10, either as single agents or in combination, were significantly attenuated in 3D vs. 2D cultures. * *p* < 0.05, ** *p* < 0.001. *t*-test. C = control, H = trastuzumab (Herceptin), T = tamoxifen, F = fulvestrant

To further explore the effects of 3D culture on drug response, we exposed BT474 or MDA361 cells to endocrine agent, trastuzumab or both agents in combination for 10 days after which cell counting were performed using a coulter counter. These experiments confirmed the dose response studies showing that 3D growth suppressed the growth-inhibitory effects of these agents. Moreover, the growth inhibitory effects of both agents in combination were also attenuated (Fig. 4a, b).

**Fig. 5:**
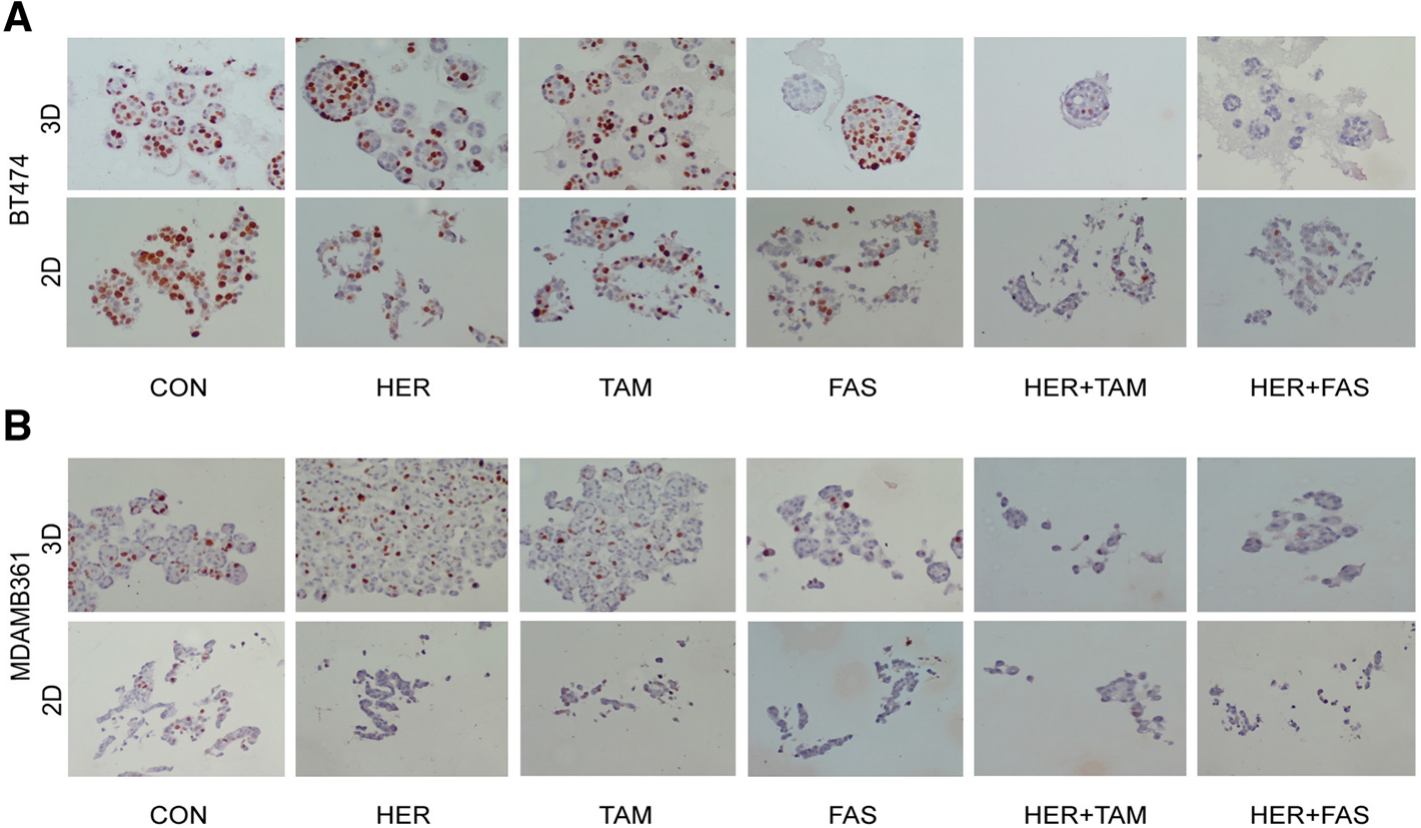
Drug-induced loss of Ki67 is suppressed in 3D culture, BT474 and MDAMB361 cells were grown for 10 days as 2D monolayers or as 3D cultures in Matrigel in the presence of either trastuzumab and endocrine (tamoxifen or fulvestrant) monotherapy or their combination treatments. CON = control, HER = trastuzumab (Herceptin), TAM = tamoxifen, FAS = fulvestrant. ICC staining of BT474 (**a**) and MDAMB361 (**b**) cells for proliferative antigen Ki67 revealed less suppression of this antigen in 3D vs. 2D culture in response to treatments

Analysis of the proliferation marker, Ki67, in cells cultured in this manner again conformed the loss of response to these therapeutics in 3D culture, showing a greater amount of staining in drug-treated, 3D cultured BT474 and MDA361 cells versus their 2D-cultuerd counterparts (Fig. 5a, b; Table 2)

**Table 2:** Ki67 H-Scores of BT474 and MDA361 cells in 2D versus 3D culture in response to endocrine agents and trastuzumab

A (BT474): Ki67 antigen staining- expressed as % control						
	C	H	T	F	H + T	H + F
3D	100	106	70	27	6	6
2D	100	33	62	25	6	2
B (MDAMB361): Ki67 antigen staining- expressed as % control						
	C	H	T	F	H + T	H + F
3D	100	75	70	25	25	5
2D	100	9	64	5	9	5

H-Scoring of immunohistochemical staining (Fig. 5) were used to determine the percent of cells positive for this antigen after drug treatment. Cells cultured in 2D had less Ki67-positive cells after drug treatment than those cultured in 3D

### Culture of breast cancer cells in 3D promotes AKT to MAPK pathway switching

**Fig. 6:**
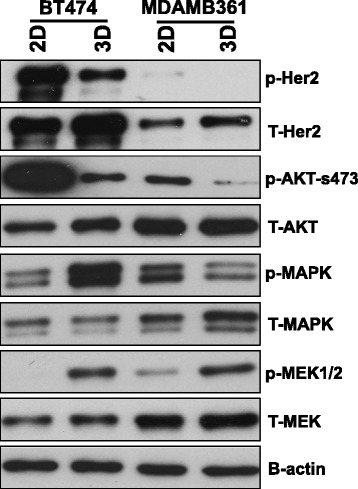
growth in 3D promotes loss of PI3K/AKT and gain in MAPK signaling. Cells were growth in 2D or 3D culture, lysed and proteins analyzed by Western blotting. 3D culture resulted in a significant loss of PI3K/AKT pathway activity and a gain in MAPK (BT474) and MEK (BT474 and MDAMB361 cells)

Having confirmed the attenuation of growth inhibitory effects of treatments in 3D versus 2D cultures we next wished to explore whether this could be explained through any significant changes in cellular signaling pathways potentially activated/suppressed in one environment compared to the other. Cell lysates from both cell lines grown in 2D and 3D were analyzed using Western blotting (Fig. 6a, b). These data revealed that growth in 3D culture resulted in a significant loss of PI3K/AKT pathway activity and a gain in MAPK signaling in both cell lines.

### MAPK pathway activity is further increased in response to treatment in 3D culture

**Fig. 7:**
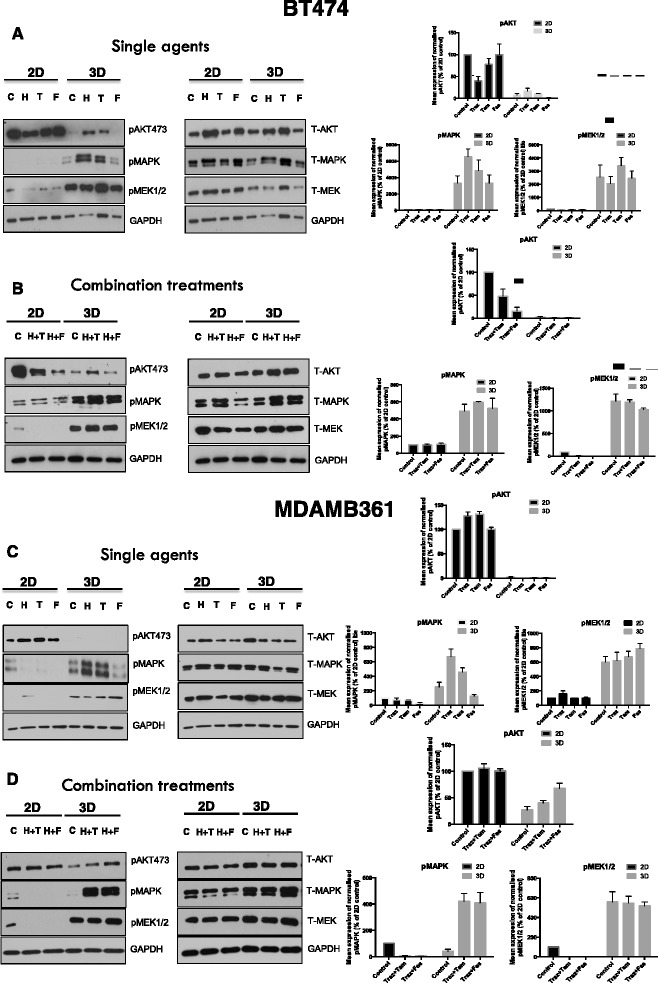
Endocrine agents and trastuzumab augment MAPK activity in 3D culture. Comparison of signaling pathway activation in 2D versus 3D culture in response to tamoxifen, fulvestrant and trastuzumab monotherapy and in combination was investigated using Western blotting with accompanying densitometry of normalized blots. For BT474, trastuzumab and endocrine treatments, either as monotherapies (**a**) or in combination (**b**), suppressed MAPK signaling in 2D monolayers in contrast to 3D culture, where MAPK activity was maintained or augmented. In the case of MDAMB361 cells, treatment with trastuzumab and endocrine agents, either as single agents (**c**) or in combination (**d**), resulted in the loss of MAPK signaling in the 2D context in contrast to 3D culture where MAPK signaling was augmented. C = control, H = trastuzumab (Herceptin), T = tamoxifen, F = fulvestrant

Given that 3D culture appeared to promote a shift from AKT to MAPK pathway, we wished to explore whether endocrine or targeted therapy had any effects on these pathways in light of the reduced chemosensitivity observed in 3D culture. Western blots and densitometry analysis (Fig. 7) of 2D and 3D cultured cell samples following endocrine agents and trastuzumab revealed that in 2D culture, monotherapy treatments had generally little effect on AKT (although suppression was observed in BT474 cells for trastuzumab) whilst combination treatments were effective at suppressing AKT activity in BT474 cells only. In contrast, the gain in MAPK activity observed in 3D culture generally appeared to be further augmented in response to single agents and, to a lesser degree, endocrine treatment and trastuzumab combined.

### Inhibition of MAPK activity in 3D culture restores sensitivity to endocrine and Her2-targeted agents

**Fig. 8:**
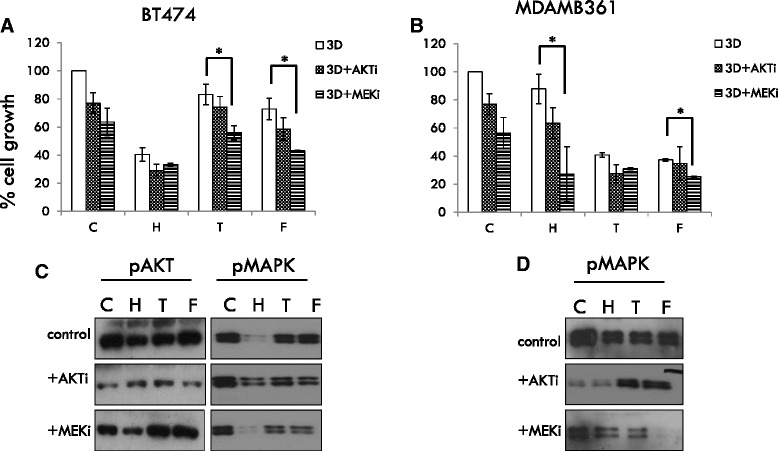
Targeting MAPK improves therapeutic response in 3D culture. 3D cultures of BT474 (**a**) and MDAMB361 (**b**) cells were treated for 10 days with trastuzumab and endocrine treatments as shown ± MEK inhibitor (U0126) or AKT inhibitor (MK-2206) and cell growth evaluated by coulter counting. Further samples were analysed for MAPK and AKT activity (**c**, **d**) by Western blotting. Inhibition of MEK significantly improved trastuzumab and endocrine response in MDMB361 and BT474 cells respectively. In both cell types, MEK inhibition but not AKT inhibition resulted in an augmentation of treatment-induced MAPK activity suppression (**c**, **d**; no data is shown for AKT in MDAMB361 cells as AKT activity was not detectable in cells in 3D culture). AKT inhibition did not improve growth suppression alone or in conjunction with trastuzumab or endocrine agent. C = control, H = trastuzumab (Herceptin), T = tamoxifen, F = fulvestrant. *p < 0.05 vs. no inhibitor

To explore whether MAPK signaling played a role in mediating therapeutic insensitivity in 3D culture, 3D cultures of BT474 and MDAMB361 cells were treated for 10 days with trastuzumab and endocrine treatments as shown ± MEK inhibitor (U0126) or AKT inhibitor (MK-2206) and their growth subsequently evaluated. For both cell lines, inhibition of AKT did not greatly affect their growth or stimulate apoptosis in 3D when used as single agents nor did AKT inhibition significantly improve the response seen with trastuzumab, tamoxifen or fulvestrant (Fig. 8a, b and Additional file 3: Figure S2). Inhibition of cell growth was modestly improved when the MEK inhibitor was used as a single agent although this was unlikely due to cell loss through apoptosis (Additional file 3: Figure S2). However, MEK inhibition improved the response to tamoxifen and fulvestrant (BT474 cells) and trastuzumab (MDA361 cells) (Fig. 8a,b). These effects corresponded to a further suppression of MAPK in the cell lines (Fig. 8c, d).

## Discussion

In this study we have investigated the impact of 3D culture on breast cancer cell sensitivity to therapeutic agents in order to better understand how drug response may be influenced by the tumour microenvironment. Our findings suggest that, for Her2+/ER+ luminal breast cancer models, their therapeutic response to the Her2 targeting agent, trastuzumab, and also to anti-ER therapies (tamoxifen and fulvestrant) is attenuated in 3D matrix enriched culture compared with 2D monolayer cultures.

2D culture systems are frequently employed to determine the effectiveness of targeted therapies in vitro although in vivo responses often fail to mirror this. For example, in vitro sensitivity to alkylating agents does not necessarily correlate with in vivo responsiveness [19], an effect that also holds true to targeted agents such as imatinib, where the degree of inhibition of proliferation obtained in vivo was substantially lower than that achieved in vitro with similar concentrations [20]. Our data and that of others thus suggest that better modelling of the in vivo environment should be considered when testing therapeutic agents in vitro. Whilst the in vivo tumour microenvironment is complex, one element of this with significant impact on therapeutic sensitivity appears to be the surrounding protienatious matrix. Indeed, a number of studies demonstrate that the tumour-matrix interaction plays an important role in governing the chemosensitivity of tumours and may also confer resistance to chemotherapeutic agents [10–14]. 3D matrix enriched models more closely mimic the in vivo conditions required for the signaling and behaviour of both normal mammary cells and also breast cancer cells and this has been confirmed by several studies. [21–24], when compared with 2D models. An in-depth proteomic analysis of Matrigel has revealed a complex and intricate mixture of proteins consisting of structural proteins, growth factors and their binding proteins as well as several other proteins of roles that are not clear in cell culture [25]. This study suggests that it will be challenging to replace Matrigel in a variety of cell culture and experimental assays due to its complexity. By using traditional 2D monolayer cultures, essential cellular functions that are present in tissues are missed and the use of three-dimensional cultures bridges this gap between cell culture and live tissue [26]. Our data here point to the importance of using appropriate in vitro models to identify the therapeutic efficacy of targeted agents in preclinical studies. Although complex models, which simulate several aspects of the tumour microenvironment, including three-dimensional culture systems, have been developed to evaluate the efficacy of therapeutic agents, these have not been adapted for routine use in high-throughput pre-clinical screening to maximize the selection of agents likely to display clinical effectiveness.

Our data points to a role for the ECM as a determinant of trastuzumab response as do other studies that demonstrate a lack of response to Her2 targeted agents when breast cancer cells are grown in 3D, laminin-rich cultures [27]. One of the underlying mechanisms here may be ECM-mediated activation of integrin signalling [12, 13] since targeting the β1 integrin, a critical mediator of laminin binding is able to restore therapeutic sensitivity to antiHer2 agents in this study. Whilst inhibition of specific integrins can improve chemotherapy response in 3D culture models, it does represent a challenge therapeutically as multiple integrin members are expressed, many of which will be involved in the tumour cells’ interaction with the diverse protein components of the ECM. Thus an alternative strategy would be to target points of convergence of signals originating from multiple integrin members. Our data suggests that inhibition of MEK signalling, known to be activated following stimulation of multiple integrin members [28–30], might represent such an approach. We show here that inhibition of MEK alone had an inhibitory effect on 3D cell growth whereas Akt inhibition did not, supporting a role for the MEK pathway in this process as we observed an increase in MEK activity when cells were grown in 3D as opposed to 2D culture. Conversely, Akt activity in 3D was reduced. Other groups have additionally demonstrated enhanced sensitivity to MEK inhibitors in 3D culture using models of triple negative breast cancer [10].

ErbB receptors are able to signal through a number of pathways including Akt and MAPK to regulate cell proliferation, migration, differentiation and apoptosis. erbB2 itself has been implicated in both activation of Akt and MAPK/MEK signalling which may reflect its dimerization state in the cell models under investigation. In our study it was interesting to note that despite erbB activity in both BT474 and MDA361 cells, only modest MAPK kinase activity was seen in monolayer culture in contrast to Akt. However, clinical studies have suggested that erbB2 and MEK signalling are linked via Pak1 (p21-activated kinase) [31], a context that involved tumour cell-matrix interactions and is supported by our 3D data. Moreover, the MEK pathway is required for maintenance of tumour cell dormancy in 3D in vitro cultures and in vivo [32]. Together these data further implicate MAPK/MEK as a pathway with relevance in the microenvironmental context.

Our findings further support other studies which suggest that the differential response to therapeutic agents seen in matrix enriched 3D cultures are a result of distinct downstream signaling cascade activation by cell-ECM interactions. Culture of both BT474 and MDAMB361 cell lines in 3D models promoted AKT to MAPK switching with a further in crease in MAPK activity observed in response to treatment with either anti-Her2 or anti-ER agents. Enhanced MEK activity is reported in in human breast cancers compared with benign breast tissue [33] whilst survival analysis has shown a positive correlation between elevated MAPK activity in primary breast cancers and decreased relapsed free survival [34]. Moreover, MEK pathway activity is associated with shortened duration of anti-hormonal response and decreased patient survival [35] supporting to our pre-clinical observations. As such, these data suggest that the MEK pathway may represent a compensatory mechanism that drives cell growth in the presence of targeted therapy. Interestingly, we observed differential sensitivity to endocrine agents between the two cell models despite them both being a Her2+/ER+ phenotype. In BT474 cells, which appeared intrinsically insensitive to endocrine agents, MAPK inhibition alongside endocrine agent resulted in a significant improvement in therapeutic response whereas no additional benefit was seen when the MAPK inhibitor was combined with herceptin. Corresponding changes in MAPK activity seemed to underlie this since only endocrine agent plus MEKi were able to further reduce MAPK activity. Moreover, these data also support others’ which suggest presence of the Her2 can limit endocrine response through MAPK signalling and modulation of ER function [36]. In the microenvironmental context, augmentation of MAPK activity, likely to occur through multiple mechanisms including integrins and erbB receptors, could further attenuate endocrine sensitivity. However the case for MDAMB361 models is less clear. Whilst also ER+/Her2+, these cells were intrinsically more sensitive to endocrine agents than BT474 models despite having lower Her2 activity in comparison. Intriguingly, treatment of MDAMB361 cells with herceptin resulted in further elevation of MAPK activity; consequently, and largely in contrast to endocrine agents, inclusion of the MAPK kinase pathway inhibitor proved beneficial in terms of inhibition of cell growth.

Thus, our data suggest that MEK inhibition in 3D culture can partially restore sensitivity to therapeutic agents and that this may be particularly useful in the context of de novo endocrine-resistant ER+/Her2+ cancers.

## Conclusions

Our data here point to the importance of the tumour microenvironment as a determinant of therapeutic sensitivity and suggests that inhibitors of MEK signalling may represent a valuable therapeutic consideration in this context. The recent development of several MEK inhibitors with potent in vivo anticancer activity have been described [37] which may hold potential for future clinical application.

## Abbreviations

AKT, Protein Kinase B; ATCC, American Type Culture Collection; ECM, Extracellular matrix; ER, Oestrogen Receptor; FCS, Foetal Calf Serum; Her2, Human Epidermaal Growth Factor Receptor 2; MAPK, Mitogen Activated Protein Kinase; MEK, MAPK/ERK kinase.

## Additional files

Additional file 1: Figure S1A. 3D ‘On-Top’ Cell culture method. A modified version of the ‘3D on-top’ assay reported by Lee et al. [17] was used for analysis of cellular growth in 3D culture and optimised for the cell lines under test here. (DOC 41 kb) Additional file 2: Figure S1B. Process of recovery of cells from 3D culture for experimental analysis. Cells were recovered from the 3D cultures for counting, immunocytochemical analysis or Western blotting as shown using a modification of a previously reported method (Arnold 2001). (DOC 52 kb) Additional file 3: Figure S2. Effects of AKT and MAPK inhibition on apoptosis in BT474 and MDAMB361 cells. Cell lines grown in 2D or 3D culture were exposed to AKT inhibitor (MK-2206) or MEK inhibitor (U0126) prior to cell lysis and Western blotting using an antibody that recognizes full length (116kDa) and cleaved (85kDa) forms of PARP, the latter form apparent upon apoptosis. Neither AKT or MAPK inhibition resulted in a significant loss of full length PARP or a corresponding gain in cleaved PARP for either cell line. In contrast, cleaved PARP was seen to increase in response to the apoptosis inducer, camptothecin, included as a positive control. (DOC 59 kb)
