# Supplementary figures and images for: 3D culture of Her2+ breast cancer cells promotes AKT to MAPK switching and a loss of therapeutic response
Source: BMC Cancer. 2016 Jun 1;16:345. doi: 10.1186/s12885-016-2377-z (PMC4888214; doi:10.1186/s12885-016-2377-z)

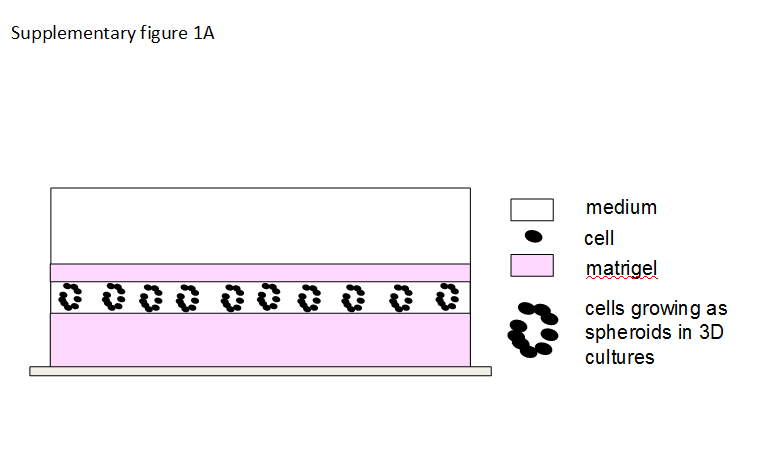

Supplement: Additional file 1: Figure S1A. — 3D ‘On-Top’ Cell culture method. A modified version of the ‘3D on-top’ assay reported by Lee et al. [17] was used for analysis of cellular growth in 3D culture and optimised for the cell lines under test here. (DOC 41 kb) [file 12885_2016_2377_MOESM1_ESM.doc]

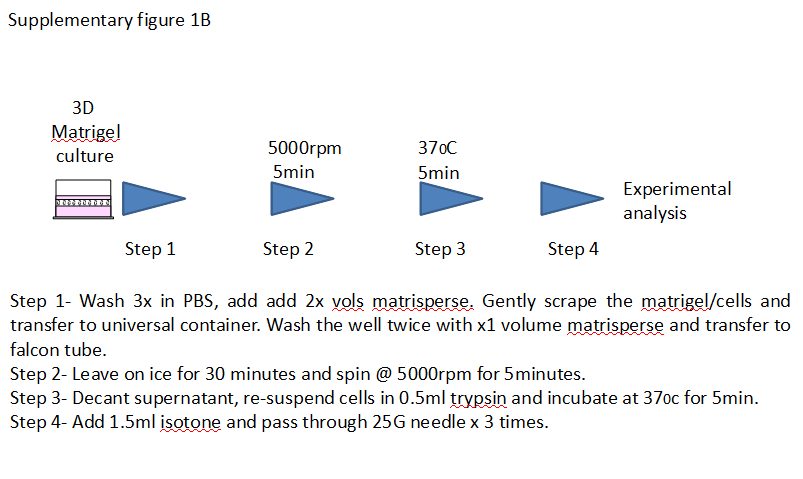

Supplement: Additional file 2: Figure S1B. — Process of recovery of cells from 3D culture for experimental analysis. Cells were recovered from the 3D cultures for counting, immunocytochemical analysis or Western blotting as shown using a modification of a previously reported method (Arnold 2001). (DOC 52 kb) [file 12885_2016_2377_MOESM2_ESM.doc]

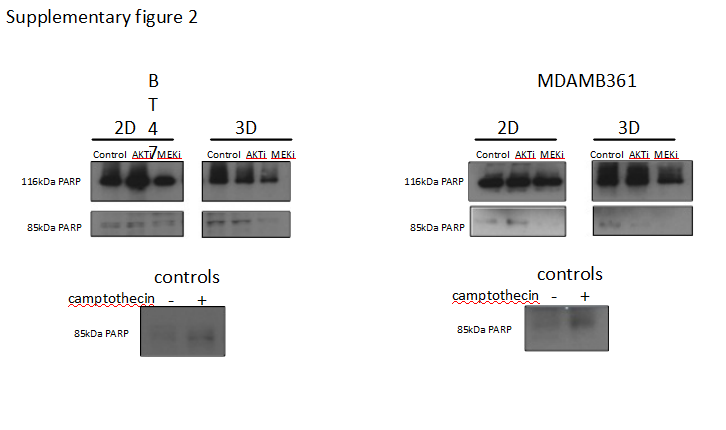

Supplement: Additional file 3: Figure S2. — Effects of AKT and MAPK inhibition on apoptosis in BT474 and MDAMB361 cells. Cell lines grown in 2D or 3D culture were exposed to AKT inhibitor (MK-2206) or MEK inhibitor (U0126) prior to cell lysis and Western blotting using an antibody that recognizes full length (116kDa) and cleaved (85kDa) forms of PARP, the latter form apparent upon apoptosis. Neither AKT or MAPK inhibition resulted in a significant loss of full length PARP or a corresponding gain in cleaved PARP for either cell line. In contrast, cleaved PARP was seen to increase in response to the apoptosis inducer, camptothecin, included as a positive control. (DOC 59 kb) [file 12885_2016_2377_MOESM3_ESM.doc]
